# Unraveling Cell Death Pathways during Malaria Infection: What Do We Know So Far?

**DOI:** 10.3390/cells10020479

**Published:** 2021-02-23

**Authors:** Camille Sena-dos-Santos, Cíntia Braga-da-Silva, Diego Marques, Jhully Azevedo dos Santos Pinheiro, Ândrea Ribeiro-dos-Santos, Giovanna C. Cavalcante

**Affiliations:** 1 Programa de Pós-Graduação em Genética e Biologia Molecular, Laboratório de Genética Humana e Médica, Universidade Federal do Pará, Belém 66.075-110, Brazil; camille.santos@icb.ufpa.br (C.S.-d.-S.); cintia.silva@ilc.ufpa.br (C.B.-d.-S.); diego.costa.santos@icb.ufpa.br (D.M.); jhully.pinheiro@icb.ufpa.br (J.A.d.S.P.); akely@ufpa.br (Â.R.-d.-S.); 2 Programa de Pós-Graduação em Oncologia e Ciências Médicas, Núcleo de Pesquisas em Oncologia, Universidade Federal do Pará, Belém 66.075-110, Brazil

**Keywords:** malaria, *Plasmodium*, immune response, cell death

## Abstract

Malaria is a parasitic disease (caused by different *Plasmodium* species) that affects millions of people worldwide. The lack of effective malaria drugs and a vaccine contributes to this disease, continuing to cause major public health and socioeconomic problems, especially in low-income countries. Cell death is implicated in malaria immune responses by eliminating infected cells, but it can also provoke an intense inflammatory response and lead to severe malaria outcomes. The study of the pathophysiological role of cell death in malaria in mammalians is key to understanding the parasite–host interactions and design prophylactic and therapeutic strategies for malaria. In this work, we review malaria-triggered cell death pathways (apoptosis, autophagy, necrosis, pyroptosis, NETosis, and ferroptosis) and we discuss their potential role in the development of new approaches for human malaria therapies.

## 1. Introduction

Malaria is a parasitic disease—caused by protozoa pathogens of the *Plasmodium* genus—that is estimated to have infected around 228 million of people worldwide in 2018, representing a risk especially for residents of developing countries in tropical and subtropical regions [1]. This is particularly relevant considering underreporting, due to diagnostic difficulties in some malaria-endemic areas. Therefore, malaria control and elimination are the central goals of the World Health Organization (WHO) Global Malaria Program (GMP); to achieve this goal, WHO recommends the administration of antimalarial drugs, but the emerging of genetic resistance in these parasites to artemisinin-based combination therapies (ACTs), the gold-standard antimalarial treatment, and the lack of an efficacious vaccine impose limitations on the progress of malaria elimination [1,2].

In the search for new methods to stem malaria, researchers have been studying intrinsic factors of the host, such as genetic profile and immunological mechanism [3,4,5]. The *Plasmodium* infection includes multiple stages, so immunity to malaria needs to be multifaceted and stage-specific [6]. The immune system has a set of strategies to fight off malaria parasites, among which is cell death. Indeed, the description of different cell death pathways underlying immune response to infectious and parasitic diseases highlighted cell death as a fundamental immunological mechanism to control parasitemia [7,8,9].

Considering the above, comprehensive knowledge about the genetic, molecular, and biochemical mechanisms of the different cell death modalities has taken a prominent position in recent advances in immune response and the design of prophylactic and therapeutic methods against malaria. This infection has been reported to induce different forms of cell death: apoptosis, autophagy, necrosis, pyroptosis, NETosis, and ferroptosis. Here, we review what is currently known about the distinct modalities of cell death of host cells during *Plasmodium* infection and the dual role of cell death in host immune protection and pathogenesis of severe malaria.

## 2. Malaria

In humans, five species of *Plasmodium* are clinically relevant: *Plasmodium falciparum*, *Plasmodium vivax*, *Plasmodium malariae*, *Plasmodium ovale* (with two sub-species: *Plasmodium ovale curtisi* and *Plasmodium ovale walikeri*), and *Plasmodium knowlesi* [10,11,12,13]. *P. falciparum* can cause the most severe complications, such as anemia, cerebral, and placental malaria [14,15]. *P. vivax* has a wider geographical distribution and, like *P. malariae* and *P. ovale*, it is associated with non-severe malaria [16,17,18,19]. However, some studies have drawn attention to the growing virulence of *P. vivax*, *P. knowlesi*, and *P. malariae*, allowing progression to severe malaria and leading to serious health problems, especially in immunocompromised patients [16,20,21]. Species that infect rodents but not humans, such as P. berghei, P. chabaudi, P. yoelii, and P. vinckei, present pathological conditions similar to the species that infect humans. Therefore, the use of these species in rodent models has contributed greatly to the advancement in immunological and pathological research of human malaria [22].

Transmission occurs primarily through the bites of infected female *Anopheles* genus mosquitoes, and occasionally via blood transfusion or vertically [23,24]. The infection initiates when the sporozoite form of *Plasmodium* enters the host dermis [25]; thus, the cutaneous lymphatic system drains some sporozoites, but most enter in blood capillaries to reach to the liver through the bloodstream [26].

In the liver, the sporozoites invade hepatocytes, where they differentiate into thousands of merozoites by schizogony process [25,27,28]. The merozoites egress back into the bloodstream to infect red blood cells (RBCs) and, in this blood stage, they undergo successive cycles of multiplication, giving rise to new parasites that infect other RBCs and initiating malaria clinical manifestations [29,30,31]. It is noteworthy that *P. vivax* and *P. ovale* present an additional challenge to malaria elimination; they can develop into dormant forms during the liver stage, known as hypnozoites, so these forms may be reactivated, causing a relapse of the disease [32].

### Malaria Immune Response and Cell Death

The innate immune system antigen-presenting cells (APCs) perform the first line of defense from the activation of pattern recognition receptors (PRRs) via recognition of *Plasmodium* pathogen-associated molecular patterns (PAMPs (DNA, RNA, and GPI anchors)) and damage-associated molecular patterns (DAMPs (heme, hemozoin, uric acid, and microvesicles)) [33,34].

In the liver stage, the detection of *Plasmodium* RNA via melanoma differentiation-associated gene 5/mitochondrial antiviral signaling protein (MDA5/MAVs) induces a type I interferon (IFN-I) response, which promotes the recruitment of cytokine-secreting cells and oxidative stress-inducing cells [35,36]. In the blood stage, this engagement depends on detection via Toll-like receptors (TLRs) of GPI anchors, parasite DNA, and DAMPs (e.g., heme, hemozoin, uric acid, and microvesicles) formed in infected red blood cells (iRBC) [37,38,39,40,41].

Upon these immune receptors’ activation, macrophages, neutrophils, natural killers (NK), T natural killers (NKT), dendritic cells (DCs), and then lymphocytes subsets readily produce pro-inflammatory cytokines—tumor necrosis factor α (TNF-α), Interferon γ (IFN-γ), interleukin-1 (IL-1), IL-6, and IL-12 [39,40,42,43,44,45,46]—oxidative-stress stimulators such as reactive oxygen and nitrogen species (ROS and RNS, respectively) [39,40,43,44], and the activation of inflammasomes [47,48], which naturally leads to an intense inflammation, high levels of oxidative stress, and, as a result, infected cells and immune cells undergo cell death [7,9,49].

Indeed, these components produced by immune cells in response to malarial infection act as activators of cell death pathways with unique genetic, biochemical, morphological, and physiological characteristics [50]. Several forms of cell death have been described as physiological adaptations to enhance immune response, performing an important role not only in eliminating *Plasmodium* but also in the pathogenesis of severe malaria.

## 3. Cell Death

Cell death (CD) is a process of metabolic disorder that leads to the loss of the cell’s vital capacity. According to the Nomenclature Committee on Cell Death (NCCD), a cell can solely be considered as dead if it presents the following criteria: irreversible plasma membrane permeabilization and/or complete cellular fragmentation [51]. Currently, the different pathways of cell death may be classified as accidental (ACD) or regulated (RCD) [51,52]. ACD is a passive process, not having any type of control mechanism; therefore, its course cannot be genetically or pharmacologically regulated [51]. In contrast, RCD has a molecular apparatus that enable genetic and pharmacological interventions [51,52].

Cell death may occur in response to different stress conditions, especially oxidative stress [53]. Research on developmental biology first identified apoptosis and autophagy as a required event for several physiological contexts in the maintenance of organismal homeostasis, so these death pathways were subclassified as programmed cell death (PCD) [54,55]. In addition to the well-established role of apoptosis and autophagy in the physiological setting, the discovery of its immunological functions, as well as the identification of other RCD pathways, has broadened the range of possibilities of cell death pathways in the pathological scenario [7,8,9,53].

Cell death and immune response have an interesting connection: while immune cells can secrete components capable of activating cell death, the death of an infected cell can release danger signals, such as DAMPs, which can provide immunostimulatory signals [56]. That said, several forms of cell death have been described as physiological adaptations to enhance immune response, performing an important role not only in clearing *Plasmodium* parasitemia, but also in the pathogenesis of severe malaria. In the next sections, we will characterize and highlight the main types of cell death in this context.

### 3.1. Apoptosis

**Figure 1:**
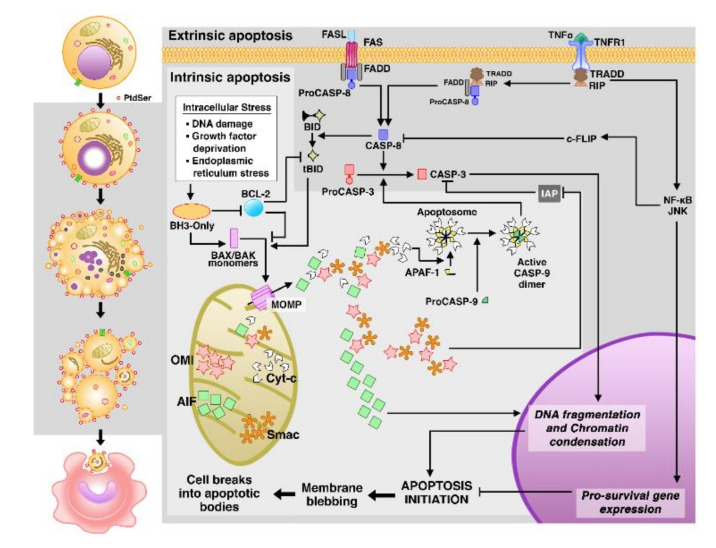
Morphological and molecular representation of apoptosis. On the left side of the figure (white background), apoptosis is morphologically characterized by alterations frequently observed such as cell shrinkage, nuclear fragmentation, membrane blebbing, and the formation of apoptotic bodies in vitro. In vivo apoptotic cells usually expose phosphatidylserines (PtdSer) in their outer membrane which function as a signal for efferocytosis by phagocytes cells. On the right, the general scheme of extrinsic and intrinsic apoptosis pathways. In the extrinsic pathway, death receptors are activated by external stimuli and, in the intrinsic pathway, mitochondrial stress stimuli occur. AIF: apoptosis-inducing factor; cFLIP: cellular FLICE inhibitory protein; Smac: Second mitochondria-derived activator of caspase; OMI/HTRA2: High-temperature requirement A2; JNK: Jun N-terminal kinase.

Apoptosis is the best-described form of PCD, and occurs in response to a wide range of physiological and pathological stimuli via two major signaling pathways: extrinsic or death receptors (DRs) and intrinsic or mitochondrial apoptosis, both resulting in activation of the caspase (CASP) cascade [55,57] (Figure 1).

External stimuli such as extracellular stress and cellular immunity activate the extrinsic pathway by providing cytokines of the TNF family, which act as death ligands, namely: FAS-ligand (FASL), TNF-α, or TNF-related apoptosis-inducing ligand (TRAIL) [58]. These death ligands interact with their corresponding DRs, known as Fas cell surface death receptor (FAS), TNF-receptor 1 (TNFR1), and TRAIL receptor (TRAILR) [58].

Apoptotic signals transmitted by death receptors engage the following adapter proteins: Fas-associated death domain (FADD) in FASL/FAS and TRAIL/TRAILR interactions [59]; and TNFR1-associated death domain (TRADD) in the TNF-α/TNFR1 interaction [60]. FADD mediates the recruitment and activation of initiators CASP-8 or CASP-10, which are present in an intracellular death-inducing signaling complex (DISC) [61,62]. In contrast to FADD, TRADD does not directly activate initiator caspases; instead, it binds to a kinase called receptor-interacting protein 1 (RIP1 or RIPK1), to the TNF-2, 5 receptor-associated factors (TRAF2, 5), and cellular inhibitors of apoptosis proteins (cIAP) [63], thus assembling the multimolecular complex I, which can engage FADD and initiator caspases, giving rise to complex II. It may also lead to the activation of NF-κB, that, in turn, can upregulate anti-apoptotic genes, suppressing apoptosis and providing inflammatory response [63,64,65].

The regulatory center of intrinsic or mitochondrial apoptosis is regulated by the B-cell lymphoma 2 (BCL-2) family gene, which includes both anti-apoptotic (e.g., BCL-2, BCL-X, and BCL-w) [66] and pro-apoptotic members (e.g., BAX, BAK, and the integrants of the BCL-2 homology 3 (BH3-only) subfamily, which includes BID, BIM, BAD, BIK, NOXA, PUMA, HRK, and BNIP3) [67].

Stress in the intracellular environment, such as damage to DNA or cellular organelles like mitochondria [68,69] and endoplasmic reticulum (ER) [68,70], stimulates TP53-mediated transcriptional regulation of the BH3-only subfamily members, which in turn counteract the activity of anti-apoptotic genes [71,72,73]. Thereafter, BCL-2 associated X protein (BAX) and BCL-2 antagonist or killer (BAK) are activated and lead to mitochondrial outer membrane permeabilization (MOMP), the event that characterizes this apoptosis pathway [68]. MOMP enables the release of apoptogenic molecules, including cytochrome c (Cyt-c), into the cytosol [74]. In the cytosol, Cyt-c binds to apoptotic protease activating factor (APAF-1) and Procaspase-9 (ProCASP-9) to form the apoptosome, a multiproteic platform for CASP-9 activation [74,75].

In the apoptosis execution phase, the initiator caspases (e.g., CASP-8, CASP-10, and CASP-9) will cleave and activate effector/executioner caspases (e.g., CASP-3, CASP-6, or CASP-7) to carry out apoptosis [76]. Effector caspases coordinate the cleavage of various molecules; among these caspases, CASP-3 is considered the most important and its role in apoptosis includes the cleavage of the inhibitor of Caspase-activated DNAse (iCAD), so that CAD is allowed to fragment nuclear DNA, resulting in chromatin condensation, degradation of cytoskeletal and nuclear proteins, and cell disintegration in apoptotic bodies [57,77,78].

The extrinsic and mitochondrial pathways are independent, but in certain types of cells, extrinsic apoptosis can be connected with mitochondrial apoptosis, through CASP-8-mediated cleavage of the BH3-interacting domain death agonist (BID) generating the truncated BID (tBID), which translocate to the mitochondria where it activates BAX and BAK to engage in mitochondrial apoptosis, and amplifying the caspase cascade to increase the efficiency of cell death [68].

In addition to the FASL/FAS route, an altered cell may undergo cytotoxic lymphocyte-mediated apoptosis through the alternative perforin/granzyme pathway [79]. This occurs when cytotoxic T lymphocytes (CTLs) recognize specific antigens and secrete lytic proteins, perforins, and granzymes (Gzm), into the target cell [80]. Perforins are pore-forming molecules, which facilitate the entrance of GzmA and GzmB [81]. Inside the target cell, GzmB triggers the cascade of caspases [82,83] and can cause MOMP by cleaving BID [84], while GzmA acts on the cleavage of the SET complex, which is implicated in DNA repair [85].

#### Apoptosis in Malaria

**Figure 2:**
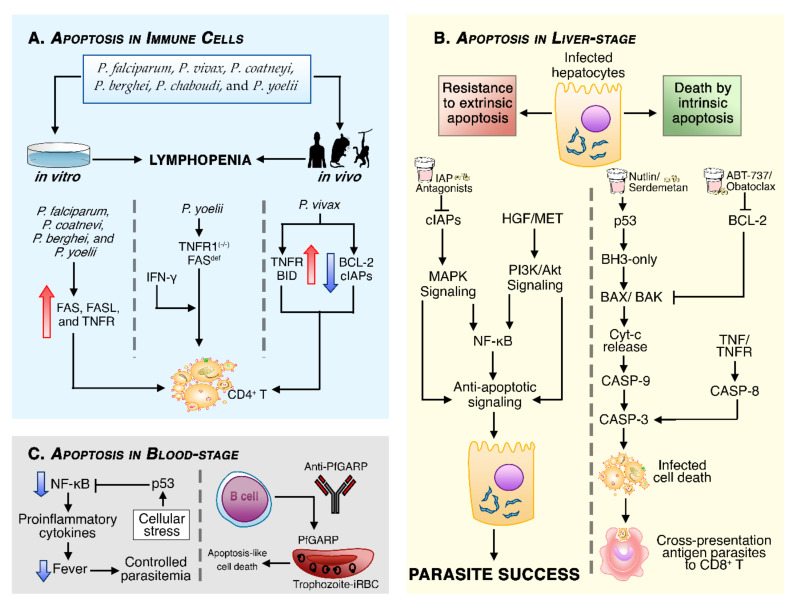
Schematic illustrations of different forms by which apoptosis is involved in malaria. (**A**) Several *Plasmodium* species were associated with extrinsic and mitochondrial apoptosis of immune system cells, but apoptosis appears to be more pronounced in T cells, leading to lymphopenia in infected humans and animals. (**B**) In the liver stage, the parasite prevents extrinsic apoptosis by activating the production of hepatocyte growth factor (HGF), which possess a strong anti-apoptotic effect in the initial phase of liver infection. In addition, the infection causes an upregulation of cIAPs by an unknown mechanism; however, infected hepatocytes are susceptible to mitochondrial apoptosis, so in the liver stage, intrinsic apoptosis plays a more prominent role in restricting the replicative niche of *Plasmodium.* (**C**) In the blood stage, p53 regulates apoptosis by suppressing NF-κB activity, and the anti-*P. falciparum* glutamic-acid-rich protein (PfGARP) antibodies kills trophozoite-infected erythrocytes by apoptosis-like cell death, conferring resistance to *P. falciparum* infection.

In the 1990s, Baldé and colleagues [86,87] were pioneers in addressing apoptosis of various immune cells of the host cultured in vitro, particularly in T cells, during *P. falciparum* acute infection [86,87] as well as in chronic asymptomatic *P. falciparum* infection [87]. In the following years, studies demonstrated apoptosis-inducing malaria-associated lymphopenia both in human malaria [88,89,90] and in animal models experimentally infected with *P. coatneyi* [91], *P. berghei* [92], *P. chabaudi* [93], and *P. yoelii* [94] (Figure 2A).

In malaria infections, there is an increase in FAS, FASL, and TNFR leading to specific and non-specific T cells death [88,91,93,94]. Despite these observations, apoptotic deletion of parasitic-specific CD4+ T cells occurred even in TNFR1 knockout (KO) (TNFR1^−/−^) and in FAS-deficient mice infected with *P. yoelii*, so researchers thought that the fate of T cells was partly defined by an IFN-γ-mediated role [95]. This hypothesis was strengthened by the association found between high levels of IFN-γ with immune splenic cell death [96,97] and IFN-γ^−/−^ mice displayed reduced splenic cell death [96]. High levels of plasma TNF and the increase in TNFR and BID gene expressions have also been demonstrated, while anti-apoptotic genes BCL-2 and IAPs were downregulated in *P. vivax*-infected patients, indicating that malaria infection induces apoptosis in CD4^+^ T cells by both extrinsic and intrinsic apoptosis [89].

Furthermore, apoptosis is involved in the depletion not only of blood DCs during *P. falciparum* [98,99] and *P. vivax* malaria [99], but also of splenic macrophages, B cells, DCs, and NK in rodent malaria infection [93,96]. The death of host immune cells might restrict the specific response to *Plasmodium* antigens; therefore, apoptosis became established as a modulator of the host’s immune response. However, we still do not fully understand the molecular details and physiological significance of activation-induced cell death (AICD) in immune cells [95,97,98,100]. Does it occur to prevent an exacerbated inflammatory response and maintain immune tolerance? Or is it a strategy developed by the parasite to subvert immune response? Although these questions remain to be answered, apoptosis in infected cells has shown to be a promising mechanism for removing parasitized cells [101,102,103,104,105,106].

The death of infected *P. berghei*-hepatocytes is independent of CTLs-mediated apoptosis either via FASL/FAS or perforin/granzyme [107], even under treatment with TNF [108] and Jo-2, a FAS-activating antibody, respectively [103]. Indeed, malaria parasites evolved mechanisms to shape apoptosis in the host cells, in order to promote their own survival and complete their growth before going to the blood-stage infection [100,108,109]. The molecular tools of resistance to extrinsic apoptosis include the hepatocyte growth factor (HGF), released by damaged hepatocytes and in overexpression of the MET gene, the HGF receptor [110] (Figure 2B). The HGF/MET signaling activates anti-apoptotic signals through PI3-kinase/AKT and, to a lesser extent, the mitogen-activated protein kinase (MAPK) pathway [109]. In addition, it has been shown that cIAPs are upregulated in the liver during *Plasmodium* infection, thus activating NF-κB and MAPK pro-survival signaling [104].

On the other hand, there is evidence that *Plasmodium*-infected hepatocytes are sensitive to mitochondrial apoptosis because of the oxidative stress generated in the infection [111,112]. Although the parasites are also able to modulate mitochondrial apoptosis by targeting TP53, BCL-2, and BAD genes [105], the expression of anti-apoptotic and pro-apoptotic genes continued to favor mitochondrial apoptosis, while the expression of the FAS and CASP-8 genes remained downregulated [111].

In order to modulate infected cell factors, Kaushansky and colleagues [103,105] revealed that pharmacological interventions to increase levels of p53 (Nutlin-3) and block BCL-2 family activities (ABT-737 or Obatoclax) selectively lead to mitochondrial apoptosis of *P. yoelii*-infected hepatocytes, reducing liver stage burden and cell susceptibility to sporozoite infection. In addition, BID^−/−^ and TP53^−/−^ mice presented considerably higher liver parasitemia compared to wild-type mice and super-TP53 mice [104]. Similar evidence was found in mice with *P. falciparum*-infected humanized liver when treated with Serdemetan, another p53 agonist, and BCL-2 chemotherapeutic inhibitors [106].

Even though infected hepatocytes are resistant to extrinsic apoptosis, genes from this pathway can also be included as targets in the search for host-based therapies [104]. Aiming for new antimalarial routes, Ebert and colleagues [104] demonstrated that the administration of IAPs’ antagonists (Birinapant and LCL-161) promoted TNF-mediated apoptosis, clearance of *P. berghei* liver infection, and induction of immunity.

As apoptotic bodies are rapidly phagocytized by macrophages and DCs through efferocytosis, apoptosis has been widely considered immunologically silent or tolerogenic [113,114]. However, further investigations pointed out that apoptotic bodies might contain pathogen antigens [56,115]. Accordingly, a study demonstrated that the efferocytosis of *P. yoelii*-infected hepatocytes that underwent apoptosis exposes parasite antigens to DCs, allowing the translating of innate response to CD8^+^ T cells-mediated adaptive response [102].

In the blood stage, high expression of p53 again appears as a protective factor against severe forms of malaria [116], being important not only to mitochondrial apoptosis, but also to limiting NF-κB-dependent proinflammatory cytokine induction [116]. Recently, a proteomic analysis of the malaria blood stage identified PfGARP as a parasite antigen, expressed in the early trophozoite stage [117]; the authors identified naturally acquired anti-PfGARP antibodies in the plasma of individuals resistant to malaria and these antibodies recognize the antigens in the surface of infected erythrocytes and direct the intra-erythrocytic parasites’ apoptosis-like cell death [117] (Figure 2C).

Although the above-mentioned studies displayed the potential of apoptosis in eliminating parasites, we should not ignore the possible adverse effects caused by imbalanced apoptosis, leading to severe complications in malaria [118,119,120,121]. It is well-known that erythrocytes are less susceptible to apoptosis as they lack mitochondria and nuclei, but they undergo a form of PCD named eryptosis, a common process upon *Plasmodium* infection [122]. The extension of this process to non-parasitized RBCs in the late phases of infection contributes to malarial anemia [118].

In placental malaria (PM) infections, genetic apoptotic markers indicate that continued exposure to malaria-induced oxidative stress leads to extensive mitochondrial apoptosis, causing placental disorder in the maternal–fetal interface and the impairment of fetal development, the main consequences being low birth weight and fetal death [119,120]. In cerebral malaria (CM), it was found that biochemical and morphological markers of apoptotic activities are involved in the degeneration of brain cells [121], mediated especially by heme/hemozoin-induced oxidative stress [123] and CD8^+^ T cells, which generate apoptosis in brain cells via granzyme/perforin processes [124].

### 3.2. Autophagy

Autophagy is a highly regulated and evolutionarily conserved mechanism that results in the lysosomal degradation of damaged cellular constituents and organelles with subsequent recycling of components of the degraded material, for intracellular clearance of potentially dangerous components in eukaryotic cells, to maintain cellular homeostasis in periods of nutrient starvations or stress [125,126,127]. In general, autophagy occurs at basal levels and works for cell survivors, but, under many insults, the autophagic machinery can change the cell’s fate and lead to cell death [128].

There other types of autophagy described: (1) microautophagy, which comprises the lysosomal invagination of cytoplasmic entities for further degradation [129]; and (2) chaperone-mediated autophagy (CMA), in which the cytosolic chaperone HSPA8/HSC70 recognizes KFERQ-containing proteins and targets them to lysosomal-associated membrane protein 2 (LAMP2) across the lysosomal membrane without the formation of autophagosomes [130,131]. This section focuses on autophagy (hereafter, macroautophagy), a complex process regulated by a set of autophagy-related genes (ATG) and divided into several phases, including initiation, nucleation, elongation, fusion, and degradation [125,131].

**Figure 3:**
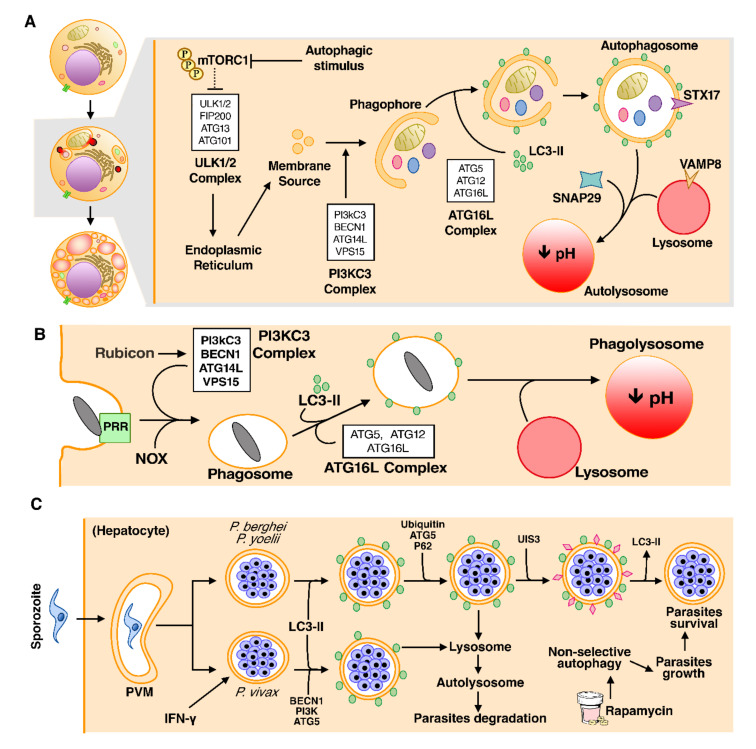
The different forms of autophagy can either act as a defense mechanism of the host or enhance the growth of the parasite. (**A**) The macroautophagy pathway, the non-selective form of autophagy. On the left side of the figure (white background), morphologically, autophagic cell death presents no changes in cell size, but is characterized by the accumulation of autophagosomes. On the right, the molecular mechanism of macroautophagy. (**B**) The LC3-associated phagocytosis (LAP) pathway, an unconventional form of autophagy that has some particularities, like the formation of a single membrane vesicle. (**C**) Different faces of autophagy in malaria infection. Host’s autophagic machinery recognizes parasitophorous vacuole membrane (PVM) and initiates the LAP-like pathway to degrade parasites. However, the *Plasmodium* relies on the UIS3 protein to remove LC3-II from the PVM and prevent LAP-like autophagy. In addition, pharmacological induction of autophagy induces the non-selective autophagic cell death of uninfected hepatocytes, which contributes to the growth of parasites.

In viable cells, the nutrient-sensitive kinase mTOR (mammalian target of rapamycin) inhibits autophagy via phosphorylation of autophagy initiators ULK1 or ULK2 and ATG13, but under autophagic stimulus, mTOR is inhibited, allowing the formation of the ULK1/2 complex or preinitiation complex, which includes ULK1/2, ATG101, ATG13, and FIP200 [132,133,134,135]. The class 3 phosphatidylinositol kinase (PI3KC3) complex—involving Beclin-1 (BECN1), vacuolar protein sorting 15 (VPS15), ATG14L, and PI3KC3 (also known as VPS34)—responds to upstream signals of phosphoregulation of the ULK1/2 complex to generate phagophore nucleation [136,137] (Figure 3A).

The biogenesis of autophagosomes, double-membrane vesicles, initiates from the phagophore elongation followed by the closure of the edge of the membranes around the cytoplasmic material to be degraded [138,139]. Phagophore elongation requires the activities of two ubiquitin-like conjugation systems, comprising: (i) The ATG5–ATG12–ATG16L complex, which is assembled by the ligase action of ATG7 and ATG10 [140]; and (ii) LC3-PE (LC3-II), generated after the lipidation of phosphatidylethanolamine (PE) to LC3, a process facilitated by the ATG5–ATG12–ATG16L complex [141]. LC3-II drives the growth and closure to form the complete autophagosome [141]. The fusion of autophagosome with lysosome, to mature both structures in the autolysosome, is regulated by the SNARE complex, involving Syntaxin 17 (STX17)—located on the outer membrane of autophagosomes—synaptosomal-associated protein 29 (SNAP29), and vesicle-associated membrane protein 8 (VAMP8) on the lysosome outer membrane [139,142]. The autolysosome has a microenvironment rich in acid hydrolase, responsible for the degradation and recycling of the contents of autolysosomes, such as cargo molecules [143].

Alternatively, a cell can undergo autophagy through a non-canonical pathway, known as LC3-associated phagocytosis (LAP), in which LC3-II directly drives the fusion of the autophagosome to the lysosome, without the assembly of autophagy core machinery. Instead, LAP requires NADPH oxidase 2 (NOX2), which mediates ROS production, and Rubicon, a protein responsible for recruiting the PI3KC3 complex to internalize invading agents [144], so that LAP have immunological functions to protect the host against infections [142] (Figure 3B).

#### Autophagy in Malaria

Studies that explore malaria infection have analyzed the autophagy aspects in both the parasite and the host [145]. Concerning the parasites, in silico and in vitro/in vivo approaches have pointed out some characteristics inherent to autophagy in *P. falciparum*. For instance, these parasites lack kinase genes that sense nutrient deprivations and initiate autophagy, but conserved the *P. falciparum* ATG8 (*Pf*ATG8) gene [146]. The *Pf*ATG8 gene is expressed in all stages of human infection and functions as a human LC3 homologous for the autophagosome formation [147,148], which allows the degradation of hemoglobin and other nutrients critical for parasite development [146,149], and even supports *P. falciparum* survival in periods of moderate starvation in intraerythrocytic infection [150].

As for the host, the first evidence of autophagic structures in liver parasitism by *P. falciparum* and *P. vivax* was reported in 1969 by De Brito and colleagues [151]. Curiously, this happened almost 30 years before apoptosis, which became the most studied death pathway in this context, while only a few studies have investigated autophagic cell death in the biology of malaria infection since then. Notably, canonical and non-canonical autophagy that act on parasite development and the defense of host cells, respectively, have been reported [152,153]. It is well-known that, in the liver stage, parasites reside within the parasitophorous vacuole (PV), which shields *Plasmodium* from an immune response, but the host’s autophagic machinery can recognize PVM; therefore, autophagy is currently considered an immune cytosolic defense [142,143].

In general, during infections, the target pathogen is first ubiquitinated to then initiate xenophagy through autophagy receptors such as p62, NBR1, and NDP52, after being labeled with LC3-II [153]. Intriguingly, in *P. berghei* infections, it has been reported that this process is inverted, initially occurring with the recognition of PVM by LC3-II, followed by the ubiquitination and binding of autophagy receptors, which leads to direct degradation of parasites into autolysosomes [154]. In studies with this species, markers indicative of LC3-II incorporating in PVM have been identified, but not the activation of BECN1; however, the deletion of the ATG5 gene led to the impairment of autophagy and parasites growth in hepatocytes, suggesting that the elimination of parasites occurred through a process similar to LAP (LAP-like) [155,156].

Moreover, Walker and colleagues [156] demonstrated that LC3-II incorporation into PVM is dependent on ATG5 functions but not on the ULK1/2 initiation complex, following a LAP-like pathway. In *P. vivax* infection, the stimulation of IFN-γ to enhance the selective LAP-like process in infected hepatocytes is needed, supporting the protective role of LAP in the host’s antimalaria immunity [157], which is termed as *Plasmodium*-associated autophagy-related (PAAR) response [158].

Despite the proven restriction of parasite replication by autophagy, evidence has shown that parasites can hijack the LAP pathway for their own benefit [155,159,160]. Studies conducted by independent groups [161,162] have demonstrated that autophagy subversion in sporozoites is furnished by the upregulation of *Plasmodium* activity in infective sporozoites 3 (UIS3), a transmembrane protein that associates with LC3 on the PVM in a protein–protein intersecting to remove LC3, contributing to parasite development within hepatocytes. This explains why many parasites manage to escape autophagy, even after recruiting LC3 and treatment with rapamycin, an autophagy inducer [160]. Furthermore, inducing autophagy with rapamycin leads to the non-selective canonical autophagy of hepatocytes, providing nutrients for parasite growth [160] (Figure 3C).

This alteration in autophagic profile does not seem to be exclusive of the liver stage. In human PM, the autophagy-associated genes *ULK1*, *BECN1*, *LC3*, and *mTOR* were downregulated [163,164], and evidenced an autophagy dysregulation which could be directly associated with PM immunopathology due to higher parasitemia [163,165] and poor pregnancy outcomes because of a reduction in placental nutrients uptake [164]. To date, few studies have investigated the role of autophagy in parasite biology and its effects on malaria pathophysiology. Nevertheless, the recent discovery of autophagy-specific mechanisms in both malaria infection progression and host response arouses the interest of autophagy as a target for vaccine improvement and therapeutic interventions [145,148,162].

### 3.3. Necrosis

Unlike apoptosis and autophagy, necrosis is lytic cell death, which is traditionally described as a form of ACD (accidental necrosis, AN) [51]. However, regulated forms of necrosis (regulated necrosis, RN) have recently emerged, of which only pyroptosis, ferroptosis, and NETosis have been well-described in malaria so far [48,166,167]. AN and RN share some morphological characteristics, including cell swelling and the loss of membrane cellular integrity accompanied by the release of DAMPs to the extracellular environment, with immunological repercussions [52]. Despite this, each RN pathway has its own genetic and biochemical characteristics, triggering inflammatory reactions to different degrees [53]. In the following subsections, we will explore the RN forms that have been described in malaria.

### 3.4. Pyroptosis

**Figure 4:**
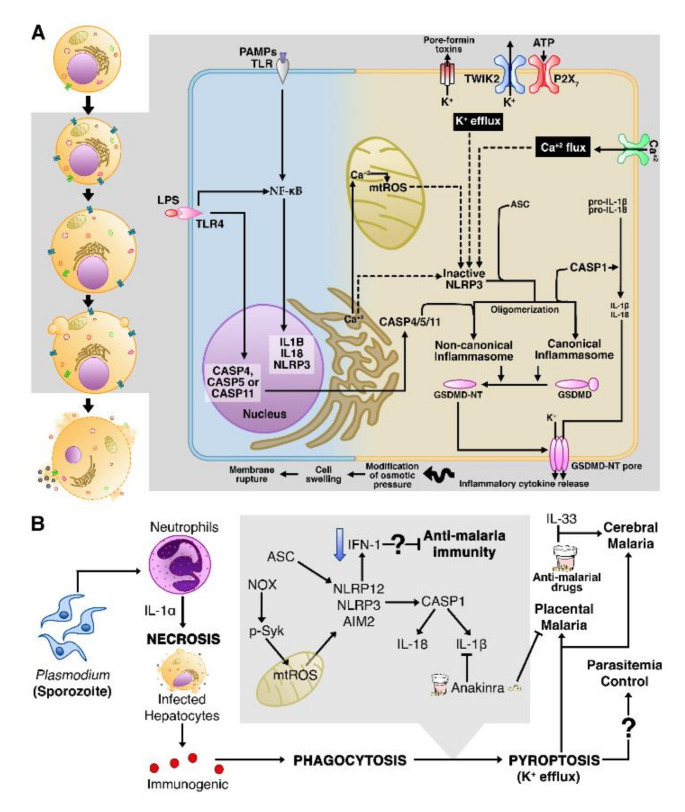
Accidental necrosis and pyroptosis act together in the pathogenesis of severe malaria. (**A**) A simplified overview of the canonical and non-canonical pathways of pyroptosis. On the left side of the figure (white background), as morphological characteristics, pyroptotic cells display cell swelling, the formation of pyroptotic bodies, and plasma membrane disruption. On the right, the molecular events of pyroptosis. (**B**) Upon *Plasmodium* infection, the IL-1α-dependent cells’ necrosis is accompanied by the release of immunogenic contents that contribute to the assembly of inflammasomes, by a mechanism to be determined. Considering that inflammasomes participate in CM and PM immunopathology, some drugs are being developed to inhibit the expression of genes involved in pyroptosis. TWIK2: two-pore domain K^+^ channel; P2 × 7: P2X purinoceptor 7.

Initially, pyroptosis was described as an alternative form of apoptosis, since both involve caspase activation [168]. However, with the identification of a highly lytic nature and other characteristics that differentiate it from apoptosis and AN, pyroptosis was recognized as a type of RN [169,170]. In general, pyroptosis plays an important role in the clearance of intracellular pathogens, through canonical or non-canonical inflammasome pathways, and subsequent processing of proinflammatory cytokines IL-1β and IL-18 [50,170] (Figure 4A).

The canonical pyroptosis initiates when TLRs recognize a range of PAMPs and/or DAMPs, thus activating NF-κB, which transcriptionally regulates the expression of genes encoding pro-IL-1β, pro-IL18, and a member of the NOD-like receptor (NLR) family, NLRP3 [171]. NLRP3 is the most studied cytosolic immunologic sensor [172], but other sensors have also been identified as participating in pyroptosis, such as other members of the NLR family (e.g., NLRP1 and NLRC4), absent-in-melanoma 2 (AIM2)-like receptors (ALRs), and pyrin [171,173].

NLRs detect “danger signals” of host or intracellular pathogens and recruit CASP-1 via apoptosis-associated speck-like protein containing a CARD (ASC), an adaptor protein, to trigger the assembly of inflammasomes [50,174]. Other factors that alter cellular homeostasis such as dysregulated mitochondrial ROS (mtROS) [175] and imbalance in ion flux (e.g., intracellular Ca^2+^ and K^+^) are also well-established activators of inflammasomes [173,176]. CASP-1 plays a key role in DNA fragmentation, generation of bioactive forms of IL-1β and IL-18, as well as the cleavage of gasdermin D (GSDMD); thus, the N-terminal portion of GSDMD (GSDMD-NT) binds to the plasma membrane to form pores that will allow the release of immunogenic contents, including IL-1β, IL-18, and DAMPs, triggering an intense inflammatory reaction [177].

In non-canonical pyroptosis, Gram-negative bacterial lipopolysaccharides (LPS) activate human CASP-4, CASP-5 [178], and mice CASP-11 [179], to directly cleave GSDMD and this induces pyroptosis [178,179]. CASP-4 and CASP-5 do not process pro-IL1β and pro-IL18, but GSDMD-NT activates NLRP3 inflammasome to maturate IL-1β and IL-18 through CASP-1 activity [178,179]. Both canonical and non-canonical pyroptosis are morphologically characterized by the modification of osmotic pressure (caused by pore formation), which leads to cell swelling and, ultimately, to membrane rupture and cell death [170].

Recently, a crosstalk between pyroptosis and other forms of cell death has been revealed. Wang and colleagues [180] therapeutically induced cancer cells to apoptosis, but instead of executing apoptosis, CASP-3 cleaved GSDME, leading to pyroptosis. Another study observed cleavage of GSDMD and GSDME during CASP-8-mediated pyroptosis in *Yersinia*-infected macrophages [181]. The inverse is also true; Taabazuing and colleagues [182] demonstrated that, with the lack of GSDMD in the canonical pathway, CASP-1 causes activation of CASP-3 and 7, and consequently, the execution of apoptosis. Unexpectedly, the cleavage of GSDMD by CASP-11 is involved in the release of neutrophil extracellular traps (NETs), formed during NETosis [183]; the release of NETs can provide the immunostimulatory molecules necessary to promote pyroptosis [184].

#### Necrosis in Infected Cells Can Trigger Pyroptotic Cell Death

An important understanding is that excessive activation of immunity in malaria leads to a proinflammatory cytokines storm with a dual role in the host: on the one hand, it works to control parasites replication; on the other hand, it causes damage to organs and tissues [33]. Much of the liver damage in malaria is a consequence of the high oxidative stress induced by heme/hemozoin and by inflammatory neutrophil infiltrate mediated by NF-κB [185]. Indeed, these cells injuries were associated with necrotic lesions in the kidneys of *P. vivax*-infected patients [186] and the liver of *P. falciparum*-infected patients [187].

In experimental *P. chabaudi* infection, necrosis in hepatic cells proved to be dependent on IL-α secreted by neutrophils [188]. In addition, an increase in IL-1α as a consequence of somatic variants, such as the *IL1A* gene single nucleotide polymorphism (SNP) rs17561, has been implicated in higher risk of severe malaria [189]. Notably, necrosis yields a variety of DAMPs and other immunogenic contents, which resulted in NLRP3 inflammasome activation, evidencing the interplay between AN and pyroptosis in the context of malaria infection [188] (Figure 4B).

The aforementioned data corroborate previous studies that determine some important events for inflammasome assembly with subsequent pyroptosis after malaria infection. Investigations in *P. chabaudi*-infected mice and *P. vivax* symptomatic malaria human patients revealed the high expression of the pyroptotic genes *CASP-1* and *IL-1β* in splenic macrophages and DCs, as well as in peripheral blood monocytes, as a result of ASC-dependent NLRP3/NLRP12 inflammasome assembly, which contributes to pyrogenic cytokines release and strong inflammation, a characteristic of malaria [48].

It has been reported that both the host’s infection-derived hemozoin [47,190,191,192] and uric acid [192,193] are inductors of NLRP3 pyroptosome. Although the low frequency in the assembly of AIM2 was noted in the study conducted by Ataide and colleagues [48], the inflammatory properties of hemozoin are boosted when it bound to plasmodial DNA and, thus, the complex hemozoin/plasmodial DNA in iRBCs can also activate AIM2 [47]. Mechanistically, pyroptosome activation in malaria infection involves phagocytosis of hemozoin and heme, an oxidative burst caused by the generation of NADPH oxidase (NOX) complexes and mtROS, considering that the phosphorylation of spleen tyrosine kinase (Syk) is necessary for mtROS generation [190,194] and K^+^ efflux through P2X7 channel [191], but it is independent of lysosomal damage [194].

Pyroptosis is widely accepted as a host advantage against infections [8,170,195], but there is controversy about the pathophysiological roles of pyroptosis in malaria. For instance, it has been experimentally demonstrated that pyroptosis does not contribute to CM [196]; adversely, in other studies, reduction in parasitemia was no longer observed upon pyroptosome overactivation [145,146], whereas NLRP3-deficient mice had higher survival rates and were more resistant to CM [145,146].

The work of Gazzinelli’s group used genetic and molecular approaches to demonstrate that CASP-8, CASP-1, mouse CASP-11, and its human ortholog CASP-4 were upregulated in monocytes from malaria patients and mice malaria models [197]. This was the first work to describe non-canonical pyroptosis activation in malaria, in a TLR4-independent process, although its role in the pathogenesis of malaria is more limited than the canonical pyroptosis [197]. Furthermore, they shed light on CASP-8’s complementary role in activating pyrogenic cytokines, such as IL-1β and TNF-α, and cleavage of GSMD, considering that the animals with combined deficiencies of CASP-8/1/11 or CASP-8/GSDM-D presented better life expectancy and were resistant to CM pathogenesis [197]. Moreover, in placental *P. falciparum* and *P. berghei* infections, the expression of the NLRP3/AIM2–CASP-1–IL-1β axis led to pregnancy complications in human and murine PM [198]. Yu and colleagues [199] showed that inflammasomes may have antimalaria immunity role, by negatively regulating IFN-I cytokines signaling, which may partly explain why mice with deficiency in different inflammasome genes (*AIM2*^−/−^, *NLRP3*^−/−^, *CASP-1*^−/−^, and *IL1R*^−/−^) were resistant to lethal *P. yoelii* infection and severe malaria outcomes.

Although a role for NLRP1 in the pathogenesis of malaria has not yet been determined, one study of genetic polymorphisms suggests that SNPs (rs1215022, rs2670660, and rs11651270) in the *NLRP1* gene are associated with malaria severity in Brazilian Amazonian patients with *P. vivax* [200]. In this work, the authors also suggest an important role for *IL-1β* (rs1143634) and *IL-18* (rs5744256) SNPs in shaping malaria clinical outcomes [200].

Exploration of the pharmacological blockage of pyroptotic genes is of interest to avoid severe malaria. A recent report has demonstrated that immunotherapy with IL-33 along with antimalarial drugs treatment selectively inhibited the formation of NLRP3 and the release of IL-1β in microglia and intracerebral monocytes of mice infected with *P. berghei*, thus reestablishing brain homeostasis and protecting it from neuroinflammation [201]. Certain chemotherapeutic drugs had some success in inhibiting CASP-1 activity (YVAD-FMK) [190] and the IL-1β pathway (Anakinra)—the latter has protected against severe complications in PM [198]. Taken together, these investigations improved the knowledge about pyroptosis in malaria pathogenesis, but no study has assessed the role of GSMD in this disease, so further investigations are recommended to better clarify the molecular steps until inflammatory pyroptotic cell death.

### 3.5. NETosis

NETosis is a type of RN that integrates the set of strategies developed by neutrophils to combat pathogenic microorganisms [202]. In this cell death mechanism, there is a rapid extrusion of a fiber network, composed of chromatin, nuclear histones, neutrophil proteins with antimicrobial properties, and to a lesser extent, mitochondrial DNA (mtDNA); these networks are known as neutrophil extracellular traps (NETs) [203,204].

**Figure 5:**
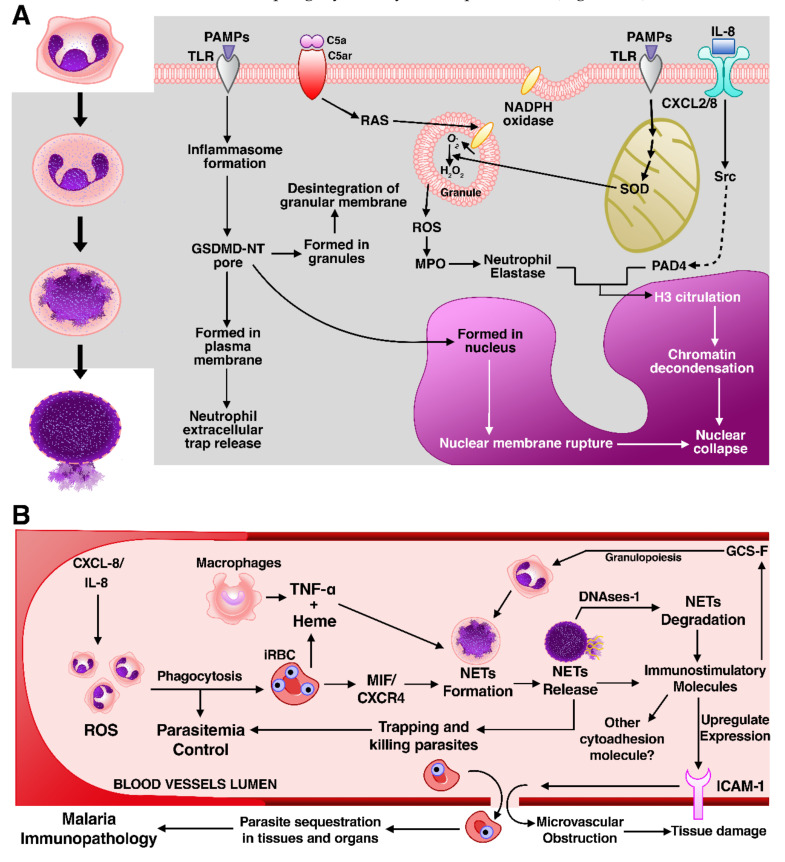
NETosis is a cause of intravascular inflammation in malaria. (**A**) A simple overview of NETosis. On the left side of the figure (white background), neutrophils’ morphological alterations during NETosis include chromatin swelling, which causes disintegration of nuclear and plasma membrane and the release of extracellular traps. On the right, the molecular mechanisms of NETosis. (**B**) After *Plasmodium* infection, neutrophils are rapidly recruited by IL-8/CXCL-8 to perform their functions, which comprise NETs formation to trap and kill parasites, but the cleavage of NETs by DNase1 facilitates NETs immunostimulatory molecules’ dissemination, causing the increase in neutrophils granulopoiesis and the upregulation of intercellular adhesion molecule-1 (ICAM-1), which promotes iRBC sequestration and inflammatory vasculature. All of these processes are considered key contributors to malaria immunopathology. C5a: Complement component 5a; C5ar: Complement component 5a receptor; RAS: renin–angiotensin system; SOD: superoxide dismutase.

Neutrophilic recognition of PAMPs and DAMPs [205] leads to ROS generation via protein kinase c (PKC) and NOX complexes’ activities [202,204,206]. ROS trigger the myeloperoxidase (MPO)-mediated activation and translocation of neutrophil elastase (NE) from neutrophil granules to the nucleus [206,207], where the combined actions of MPO and NE promote histone degradation and, consequently, chromatin decondensation [207]. NE may also degrade actin filaments in the cytoskeleton to block movement and phagocytosis by neutrophils [206] (Figure 5A).

Furthermore, histone modification and chromatin dispersion also depend on the direct activation of peptidyl-arginine deiminase 4 (PAD4) by ROS [208]. PAD4 drives the conversion of histone arginine residues in other substrates, such as ketone by citrullination [208]. This process facilitates the disintegration between DNA and histones [209], causing an expansion of chromatin. Swelling of chromatin is the major cause of rupture of the nuclear envelope and plasma membrane, allowing the release of NETs to capture and kill infectious agents [210].

Alternatively, neutrophils can release NETs in the extracellular environment through a non-conventional mechanism of NETosis. This mechanism requires factors like ROS, MPO, and NE activity, as well the destruction of neutrophils; instead, as mentioned in the subsection “Pyroptosis”, NETs are released in vesicles or neutrophil serine proteases via GSDMD cleavage [184,211]. It is important to highlight that other granulocytes are also capable of producing ETs and lead to cell death [212]. Therefore, it is still a topic of discussion among researchers on whether the correct term to adopt is NETosis or ETosis, since NETosis would be a specialized form of ETosis [213].

#### NETosis Leads to Severe Malaria

So far, NETosis is the only granulocyte RN described in malaria. Even though many details of NETosis mechanisms remain unknown, the role of this type of cell death in either protection against *Plasmodium* or tissue damage resulting in the pathogenesis of severe malaria has been brought to attention; therefore, knowledge on this relationship is gradually progressing. Neutrophils are the most abundant innate immunity cells and, differently from lymphocytes, their number generally increases in malaria infection [214,215]. These cells employ a wide range of strategies to control pathogens dissemination, including the extrusion of NETs, but these molecules also have harmful effects in host tissues [215]. Accumulating data have implicated neutrophilic mechanisms in the pathogenesis of CM [216,217], liver failure [218], lung injury [219], and PM [220].

Baker and co-workers [172] reported the first evidence of blood-circulating NET-like structures entrapping parasitized erythrocytes and free trophozoites in children with uncomplicated *P. falciparum* infection. The group proposed that the release of NETs would induce severe malaria pathogenesis in children, but it would confer immunoprotection in adults [221]. However, immunoprotection does not seem to be the only result of NETosis; for instance, the expression of NETosis markers (NE and MPO) was increased in adults with *P. falciparum*, *P. vivax*, and *P. malariae* infections, while NETosis was correlated with parasite biomass reduction, associated with progression to severe malaria [167].

Data from independent groups further support the importance of NETosis in promoting inflammation during malaria infection. *P. falciparum* and *P. berghei* stimulated neutrophils to form NETs, which damaged the alveolar–capillary barrier, facilitating the infiltration of inflammatory cells and cytokines, ultimately contributing to the establishment of malaria-associated pulmonary pathologies [175]. Boeltz and colleagues also proposed that NETosis in the intravascular space of tissues triggered inflammatory events [222].

However, the mechanism of NETosis activation and its pathophysiology in malaria became slightly clearer when Knackstedt and co-workers [223] revealed that free heme and TNF-α induced NETs formation during infection, in which NETosis required NE and PKC activity but was independent of PAD4 citrullination. Furthermore, in this same investigation, NETosis mediated vasculature inflammation by immunostimulating the emergency granulopoiesis in macrophages via granulocyte colony-stimulating factor (G-CSF) and by upregulating ICAM-1, an important receptor of cytoadhesion, which mediates the sequestration of iRBC in brain and liver microvasculature, therefore being a key mediator of cerebral malaria as well as liver and lung injuries (Figure 5B).

Rodrigues et al. [224] also brought important information to the discussion on the pathogenic activity of NETosis in malaria. In this study, differently from the previously mentioned work, by conducting an in vitro analysis of *P. falciparum*-iRBCs, the main inductor of NETs formation was the chemokine CXCR-4, being dependent on PAD4 histone citrullination and independent of MPO, NE, or ROS formation. Although both researchers have performed an in vitro analysis under similar conditions, the differences between them extend to the biological function of NETosis underlying malaria: while Knackstedt et al. did not observe changes in parasitemia but the increased in vivo susceptibility to severe malaria, Rodrigues et al. suggest that NETosis interfere in *P. falciparum* dissemination and improve host survival.

Despite controversial results, most studies agree that, in vivo, NETosis mainly presents a harmful role in the integrity of organs and tissues, whether in mice or in humans [167,219,223]. Therefore, the development of pharmacological inhibitors of neutrophils has been suggested [219,222,223], considering that, currently, there are no methods to genetically eliminate NETosis. However, we still do not know which consequences could occur from the inhibition of neutrophils in humans, given the importance of these cells in controlling *Plasmodium* infection.

### 3.6. Ferroptosis

Ferroptosis is an RN form that depends on iron and amino acid metabolisms. It is characterized by overwhelming lipid peroxidation and dysregulation of homeostatic balance maintained by glutathione peroxidase 4 (*GPX4*) gene activity, which encodes an enzyme that is indispensable for the degradation of lipid hydroperoxides [225,226].

**Figure 6:**
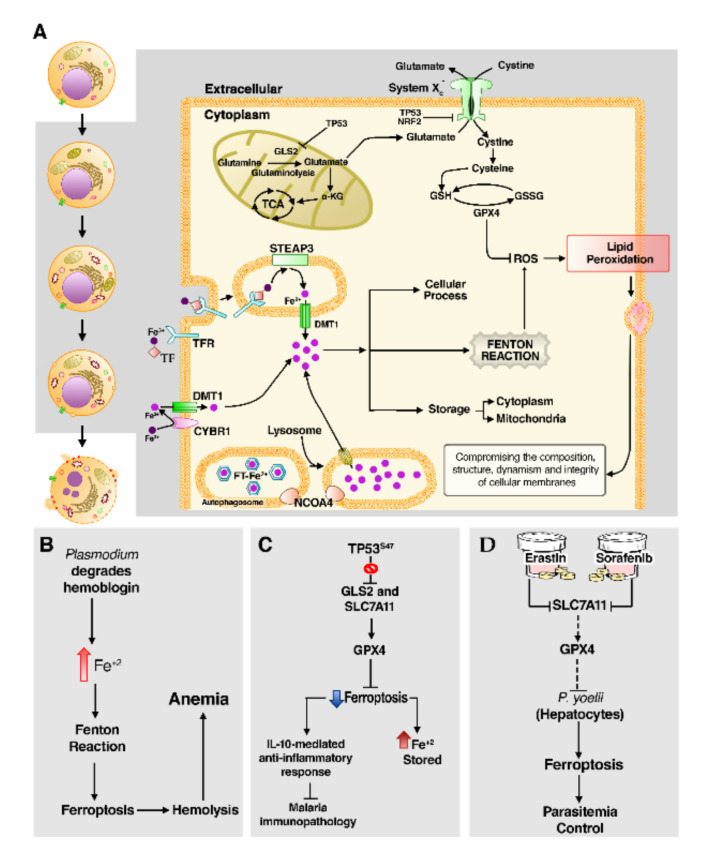
The emerging effect of ferroptosis in malaria immunity. (**A**) An overview of the ferroptosis pathway. On the left side of the figure (white background), morphologically, ferroptotic cell death is distinguished by structural changes in mitochondria like outer membrane disruption and volume reduction as well as cell rounding followed by plasma membrane rupture. On the right, the genetic regulatory routes of ferroptosis. (**B**) The consumption of hemoglobin by *Plasmodium* releases Fe^2+^ atoms that may catalyze the Fenton reaction, causing iron–lipid peroxidation and hemolysis, this process being involved in anemia. (**C**) Variant S47 of *TP53* impair ferroptosis, causing iron accumulation in macrophages, which elevates IL-10 levels and provides an anti-inflammatory response, limiting the immunopathological damage of malaria. (**D**) On the other hand, the induction of ferroptosis by the pharmacological blocking of *SLC7A11* and *GPX4* reduces *Plasmodium* liver parasitemia.

Iron is an essential nutrient for humans with clear biological functions in many cellular processes [227]. Cells absorb ferric iron (Fe^3+^) preferentially via transferrin receptor (TFR1) and, occasionally, divalent metal transporter 1 (DMT-1) absorbs ferrous iron (Fe^2+^) [228]. The Fe^3+^ form is then reduced to Fe^2+^ in endosomes by six-transmembrane epithelial antigen of the prostate 3 (STEAP3), then transported to the cytoplasm to be used as a cofactor of cellular processes or stored as cytosolic and mitochondrial ferritin (FtMt) [229]. Ferritin degradation by nuclear receptor coactivator 4 (NCOA4)-mediated ferritinophagy releases the iron required for cellular processes, but also generates ROS through the Fenton reaction, which is the oxidation of Fe^2+^ to Fe^3+^ [230,231] (Figure 6A).

The accumulation of ROS generated by excess iron reacts with lipid membranes and may reach a lethal level of lipid peroxidation [232]. In a lipidomic study, Yang and colleagues [232] demonstrated that polyunsaturated fatty acids (PUFAs) are the most susceptible lipids to iron-dependent peroxidation, evidencing that lipid metabolism is closely linked to ferroptosis. In cellular membranes, Acyl-CoA synthetase long-chain family member 4 (ACSL4) [233,234] and lysophosphatidylcholine acyltransferase 3 (LPCAT3) participate in the biosynthesis and remodeling of PUFA-containing phospholipid hydroperoxide (PUFA(PE)-OOH), required for ferroptosis progression [226].

To reduce lethal accumulation of lipid peroxidation, the cell has antioxidant systems, especially in the cysteine-glutamate antiporter system X_C_^−^, which is composed of the subunits solute carrier family 7 member 11 (SLC7A11) and SLC3A2 [235,236], as well as GPX4 [237]. Under normal physiological circumstances, system X_C_^−^ ensures proper operation of GPX4 by regulating glutathione synthesis [225,238]. The Glutaminase 2 (GLS2) catalyzes mitochondrial glutaminolysis to provide the necessary glutamate to be exchanged for extracellular cystine via system X_C_^−^ [239]. The majority of cystine is available to be converted into cysteine, the precursor of glutathione (GSH) biosynthesis, which is then used by GPX4 as a cofactor to scavenger lipid peroxidation through a reduction in lipid peroxides in the cell membrane and to suppress ferroptosis [237]. Besides that, glutaminolysis is a source of α-ketoglutarate (α-KG), thus contributing to increasing activity of tricarboxylic acid (TCA) cycle and the accumulation of mtROS, which may amplify ferroptosis by an unknown mechanism [239,240].

At the level of transcriptional regulation, TP53 increases cell sensitivity to ferroptosis by upregulating GSL2 expression [241]. This highlights a potential role of mitochondrial metabolism in the amplification of ferroptosis, but other explanations should be considered since glutaminolysis also generates glutamate, required for the synthesis of glutathione [239]. Additionally, TP53 represses SLC7A11 expression, impairing cystine import and the synthesis of GSH and GPX4 [242]. In contrast, nuclear factor erythroid 2-related factor 2 (NRF2) protects against ferroptosis by upregulating SLC7A11 expression and anti-ferroptosis amino acids [243].

#### Ferroptosis Leads to Lipid Peroxidation in Malaria

Ferroptosis is suggested to be involved in cancer and neurodegenerative diseases, but its immunological functions are still poorly understood [244]. Possibly, the role of ferroptosis in malaria infection is implicated in oxidative stress, which was generated by phagocyte-derived ROS/RNS as host protection mechanisms or through the cell infection process itself [245]. Despite the importance of ROS and RNS as components to combat parasites, their excess can cause serious damage to the plasma membrane lipids, resulting in lipid peroxidation [245].

Iron is an essential nutrient for both humans and several pathogens, thereby iron status of human hosts regulates the susceptibility and the course of malaria infection [246,247]. Since *Plasmodium* needs hemoglobin to grow and develop, these parasites invade the iron-rich environment of RBCs [247]. The heme formed during the degradation of hemoglobin contains Fe^2+^ and its liberation catalyzes the Fenton reaction [248,249].

Several studies have identified a substantial increase in lipid peroxidation in RBCs infected by different *Plasmodium* species [245,250,251], concomitantly with hemolysis and a decrease in antioxidants, including GSH [245,251]. In human malaria patients, iron-dependent lipid peroxidation was enhanced in those with malaria caused by *P. falciparum* than by *P. vivax* [251]. The dysregulation in iron homeostasis mediates the accumulation of hydroperoxides and leads to cellular inflammation and death, which takes part in malaria anemia [245,250] (Figure 6B); thus, a deeper understanding of ferroptosis effects on malaria severity is still needed.

Jennis et al. [252] found that the non-synonymous SNP rs1800371 (referred to by the authors as P47S or S47) impairs the ability of TP53 to regulate genes involved in ferroptosis, including *GLS2* and *SLC7A11*, leading to defects in ferroptotic cell death and iron accumulation. Mouse macrophages containing the S47 variant presented resistance to ferroptosis and elevated iron status, which decreased proinflammatory cytokines and increased IL-10-mediated anti-inflammatory response under exposition to malaria hemozoin [253] (Figure 6C). This indicated that prevention of ferroptosis impacts directly on the generalized inflammation associated with malaria immunopathology, so this effect may explain the fact of the S47 variant is predominantly found in Sub-Saharan Africans and individuals of African ancestry, since these individuals live in malaria-endemic regions [252,253].

Paradoxically, a large amount of iron provides the supply for *Plasmodium* development in both liver and blood stages [246,254]. In this sense, a recent work developed by Kain and colleagues [166] showed that blocking of the SLC7A11-GPX4 pathway in *P. yoelii*-infected hepatocytes directs these cells to lipid peroxidation and death via ferroptosis; the same occurs when cells are treated with Erastin and Sorofenib, pharmacological inhibitors of SLC7A11 (Figure 6D). In contrast, pharmacological induction of p53 was not able to lead to ferroptosis under NOX1 or TFR1 blockage [166]. Therefore, this result suggests that ferroptosis might play a significant role in inhibiting *Plasmodium* growth in hepatocytes, by depriving malaria parasites of iron and by the selective death of infected cells.

## 4. Future Perspectives

Currently, it is known that cell death pathways play critical roles in response to infections, given that the death of an infected cell is directly related to malaria immunopathology and the death of parasites, so these pathways are taken as inherent mechanisms of the immune system and a key process to understand host–parasite interaction. In this context, RCD pathways undoubtedly have the potential to become a new avenue for the development of preventive and therapeutic measures.

For such reasons, the study of cell death pathways in infectious diseases is a growing field, but there is still a lot to advance in the knowledge of the genetic, molecular, and biochemical mechanisms of cell death pathways triggered during *Plasmodium* infection. For instance, we know that the signaling of a specific death pathway takes place depending on different factors, such as the type of the dying cell, the species of pathogen, and the pathogen load [8]. Currently, various forms of cell death have been described in malaria, but which one is the most efficient to subvert the parasite and improve the outcome of the disease?

Apoptotic signaling is among the most common cell death pathways in malaria, although some gaps remain to be resolved in future studies. For instance, as we reviewed here, HGF/MET signaling ensures parasitized hepatocytes’ survival only at early stages of infection, which led to the idea that there is an additional mechanism that promotes cells survival at late stages. Recently, a study showed that cIAPs are upregulated during malaria infection, but how do cIAPs become upregulated in this infection? Is it *Plasmodium* that regulates cIAPs directly or indirectly? Why and how can these parasites modulate and resist extrinsic pathways, but cannot avoid mitochondrial apoptosis? In this sense, various works have pointed out cIAPs’ and BCL-2 antiapoptotic genes’ antagonists, as well as p53 agonists as potential targets for antimalarial drugs development [103,104,105,106].

So far, differently from apoptosis, little is known about the mechanisms involved in the other RCD in malaria. Regarding autophagy, more investigations are necessary to explore the aspects related to the host–parasite interaction, such as how the autophagy machinery interact with immune response and its consequences for the host and the parasites. It is also not fully comprehended how the parasite evades the host’s autophagy, as well as how the host’s machinery recognizes the infection and prevents the growth of the parasite.

We cannot disregard other forms of cell death that have been coming up as potential therapy targets for infectious diseases. Since pyroptosis and NETosis did not have the expected effect on parasitemia control, causing serious damage to tissues and organs of the host, it would be more beneficial to inhibit them rather than to stimulate their execution. Although it has been shown that ferroptosis can limit parasitemia in the liver, we still do not know much about the immune potential of this type of RCD. It would be interesting for future investigations to consider which is the best method of prevention or treatment of malaria, whether it is by neutralizing the parasite through immunologically silent apoptosis or by inducing a setting of immunological responses through immunogenic apoptosis or ferroptosis, studying what would be the potential of these RCD forms in the elimination of liver hypnozoites in people exposed to *P. vivax* and *P. ovale* sporozoites. 

Lastly, necroptosis—a form of RN mediated by RIPK1 and RIPK3—can be executed downstream of the same DRs that activate extrinsic apoptosis, particularly under FADD/CASP-8 deficiency or suppression, and can also be triggered by TLRs [53]. Whereas heme induces TLR4 activation, which can activate RIPK1/3, a role for necroptosis has been proposed, but how exactly necroptosis contributes to malaria outcome is still unknown [248]. As the role of necroptosis and some other forms of necrosis intriguingly have not yet been studied in malaria, this is a topic for future research studies.

## 5. Conclusions

Malaria is considered one of the major parasitic infections in humans, and different studies have added important insights in an attempt to develop new prophylactic methods and improve the already existing methods. In this scenario, studies on cell death pathways offer relevant information to achieve this goal, particularly RCD forms. Therefore, it is necessary to understand the unique characteristics of each cell death form and the factors that lead an infected cell to employ a certain pathway. In this sense, this review contributes to this major goal by providing the current state of research in this field, unraveling pathways and factors that are potential therapeutic targets for malaria, and recommending further approaches.
